# Evaluation of Quality of Life in Adults Following Cochlear Implantation 

**DOI:** 10.22038/ijorl.2025.89667.4004

**Published:** 2025

**Authors:** Leila Monshizadeh, Seyed Basir Hashemi, Mehdi Rahimi

**Affiliations:** 1 *Otolaryngology Research Center, Department of Otolaryngology, Shiraz University of Medical Sciences, Shiraz, Iran. *; 2 *Otolaryngology Research Center, Department of Otolaryngology, Shiraz University of Medical Sciences, Shiraz, Iran. Department of Otorhinolaryngology-Head and Neck Surgery, School of Medicine, Shiraz University of Medical Sciences, Shiraz, Iran.*; 3 *Assistant Professor of Psychology Unitec Institute of Technology, New Zealand.*

**Keywords:** Quality of life, Adults, Cochlear implantation

## Abstract

**Introduction:**

Cochlear implantation is a surgical procedure which provides the sense of hearing in patients with sensorineural hearing loss, particularly when conventional hearing aids are no longer effective. Although cochlear implantation is mostly used for children, increasing number of adults are also benefiting from this life-changing technology. As cochlear implantation can improve communication in adults, enhance quality of life and socio-emotional well-being, the primary aim of the present study is to investigate the quality-of-life improvement in adult cochlear implant recipients

**Materials and Methods:**

This quasi-experimental single-group pretest-posttest study utilized the Persian standardized version of the World Health Organization Quality of Life questionnaire (WHO-QOL-BREF) to assess quality-of-life improvements in 26 adult cochlear implant recipients with a mean age of 36.19 ± 12.71 years. The questionnaire was administered at two time points: the first month after receiving the speech processor and six months later. Data analysis was conducted using SPSS version 21.

**Results:**

A repeated-measures ANOVA was performed to examine the effects of cochlear implantation and the subsequent rehabilitation program on four quality-of-life dimensions: physical health, psychological health, social relationships, and perception of the living environment. The analysis revealed a significant main effect of time on quality of life, indicating notable improvements across all dimensions from pre- to post-treatment.

**Conclusion:**

The pre- and post-test analysis using the WHO-QOL-BREF questionnaire demonstrated a significant enhancement in the quality of life among adult cochlear implant recipients. Therefore, cochlear implantation is an effective intervention for treating hearing impairment in adults suffering from progressive hearing loss.

## Introduction

Cochlear implantation is a procedure that involves implanting electrodes to bypass damaged auditory cells in the inner ear and directly stimulate the auditory nerve. While its primary objective is to restore hearing, additional benefits include language development, improved speech perception, enhanced emotional well-being, social interaction, and better engagement in daily activities (1).

The World Health Organization (WHO) predicts that by 2050, one in four people may suffer from severe hearing loss (2), leading to cognitive impairment, social isolation, and loneliness. Cochlear implantation, along with a structured rehabilitation program, can mitigate these negative effects by enhancing auditory perception and speech recognition, thus improving communication in personal, social, and professional settings (3-5). 

It enables individuals to perceive sounds more clearly and improves speech recognition. This fosters better communication in personal, social, and professional settings. With better hearing, individuals often feel more confident in group conversations and public settings, reducing social isolation and fostering a sense of inclusion. 

Cochlear implants also contribute to safety and self-reliance by allowing users to respond to environmental sounds such as alarms and traffic signals. Additionally, they help reduce frustration, anxiety, and depression associated with hearing loss. Many recipients report an overall increase in happiness and satisfaction, improved job performance, and greater access to career opportunities through better communication and participation in rehabilitation programs. Studies suggest that treating hearing loss with cochlear implants can help mitigate cognitive decline by keeping the brain actively engaged with auditory input. Improved auditory perception strengthens relationships with family, friends, and colleagues, ultimately enriching social interactions and community bonds.

A study in 2012 evaluated health-related QoL in adults with profound post-lingual hearing loss before and after cochlear implantation using the AQoL-8D questionnaire. Findings revealed significant improvements in overall QoL, particularly in mental health, happiness, self-worth, coping, and relationships dimensions. The greatest changes were observed in the senses and self-worth dimensions (3). A research in 2018 involving 26 adults with an average of 6.6 years of cochlear implant use reported benefits in various QoL aspects. The psychological domain scored highest in the WHO-QOL questionnaire. Notably, the ability to comprehend speech over the telephone was associated with a better perception of QoL across all domains (6)(article 1).

A 2017 prospective cohort study with 61 patients found significant improvements in health-related QoL following cochlear implantation. The Nijmegen Cochlear Implant Questionnaire (NCIQ) showed significant enhancements in seven of eight subdomains, while the SF-36 reflected improvement in one of nine domains. This suggests that disease-specific measures like the NCIQ may be more sensitive in detecting QoL changes post-implantation (7).

Furthermore, a 2024 study highlighted the role of perceived social support in improving health-related QoL among cochlear implant patients. Demonstrating that individuals with greater social support experienced better QoL improvements post-implantation (8). 

According to above mentioned studies, it can be said that cochlear implantation in adults with post-lingual hearing loss leads to significant improvements in QoL, particularly in communication, psychological well-being, and social interactions.

In the same direction, the primary aim of the present study is to assess life quality in adults’ cochlear implanted patients.

## Materials and Methods

It is a quasi-experimental single group pretest-posttest study in which all 26 recent adults’ cochlear implant recipients (14 male, 12 female) with mean age of 36.19±12.71 were assessed through the brief form of WHO Quality of Life questionnaire (WHOQOL-BREF). The main reason for choosing a single-group pretest–posttest design was as following: This design allows researchers to evaluate the effect of an intervention by comparing participants’ outcomes before and after exposure to the treatment. Although it lacks a control group, it still provides valuable preliminary evidence about potential changes associated with the intervention. The pretest serves as an internal baseline, allowing each participant to act as their own control.

WHOQOL-BREF is a widely used tool for assessing quality of life across different domains. It was developed to evaluate individuals' perceptions of their position in life in the context of their culture, value systems, personal goals, and concerns. It is designed to be culturally sensitive, making it applicable across various global populations(9). The test is in four sections of physical health, psychological health, social relationships and perception of life environment which have 7, 6,3, and 8 questions respectively. The first and second questions are about every body’s overall perception of life and health. 

The validity of the WHOQOL questionnaires has been confirmed through extensive research, showing strong correlations between the questionnaire domains and the quality-of-life constructs. The WHOQOL questionnaires demonstrate high reliability, characterized by internal Consistency (high Cronbach's alpha values across domains suggest strong coherence among items), test-retest reliability and inter-rater reliability (10).

The Persian adapted version of WHOQOL-BREF questionnaire was conducted out to assess the probable improvement in life quality of adults’ cochlear implant users during the first six month of implantation. It has been evaluated for its validity and reliability within Iranian populations. Studies indicate that this adaptation maintains robust psychometric properties, making it a suitable tool for assessing quality of life in Persian-speaking individuals. Factor analyses have supported the four-domain structure (physical health, psychological health, social relationships, and environment) of the Persian version of WHOQOL-BREF, aligning with the original instrument's design. Cronbach's alpha coefficients for the Persian version of WHOQOL-BREF domains generally exceed the acceptable threshold of 0.70, indicating satisfactory internal consistency. 

However, the social relationships domain has shown lower alpha values in some studies, suggesting the need for cautious interpretation (11,12). 

To evaluate the quality of life in adult cochlear implant users, all patients meeting the inclusion criteria were selected. The youngest patient was 20 years old and the oldest was 68.83 years old. Among the participants, only two had master of sciences. However, the educational level of the rest was diploma or lower. Although the sample size was relatively small (n = 26), it accurately represents the limited number of adult cochlear implant recipients available during the study period. All adult patients who had received their speech processor by June 2024 and had no additional disabilities were included. 

According to the conducted interviews, the socio-economic status of the participants was approximately comparable, which helped minimize potential confounding variables. A sensitivity calculation shows that with n = 26 the minimum detectable standardized effect size at α = 0.05 and 80% power is approximately d ≈ 0.55 (This study has ~72% power to detect d = 0.5). 

 The uni-lateral implantation was performed for all the patients. It is necessary to remind that progressive hearing loss was the main cause of deafness in this group of patients.

All procedures performed in the present study followed the ethical standards of our university research committee (Ethic code: IR.SUMS.REC.1397.631), and with the 1964 Helsinki declaration and its subsequent amendments. The informed consent form had been obtained too. After performing the test by the first month of implantation and six months later, a repeated-measures ANOVA was conducted to evaluate the effects of cochlear implantation and its follow-up rehabilitation program on four quality-of-life dimensions. We analyzed the data using statistical software for social sciences version 21 (SPSS-21). The statistical significance was accepted at P < 0.05.

## Results

A repeated-measures ANOVA was conducted to evaluate the effect of cochlear implantation and its follow up rehabilitation program on four quality of life dimensions: physical health, psychological health, social relationships, and perception of life environment, in the first month of receiving speech processor and six months later. Descriptive statistics of the participants is depicted in table 1. According to the data mentioned in this table, the mean scores of four quality of life dimensions improved during the first six months of implantation.

**Table 1:** Means and standard deviations of quality-of-life dimensions by the first month of implantation and six months later

**measure**	**N**	**First month of implantation**		**Six months after implantation**	
		**Mean**	**SD**	**Mean**	**SD**
Physical health	26	18.56	3.13	26.73	3.96
Psychological health	26	15.03	3.03	21.23	4.65
Social relationships	26	8.84	1.54	10.80	2.11
Perception of life environment	26	24.50	5.45	28.30	6.14

The analysis indicated a significant main effect of time on quality of life, (Pillai’s Trace = 0.759, F (4, 22) = 17.363, p < .001, partial η² = .759), suggesting a notable improvement across the dimensions from pre- to post-treatment. The participants’ performance after six months of cochlear implantation is shown in table 2. 

The follow-up univariate tests showed significant improvements in all four dimensions: physical health (F = 72.381, p < .001, partial η² = .743), Psychological health (F = 39.812, p < .001, partial η² = .614), social relationships (F = 23.827, p < .001, partial η² = .488), and perception of life environment (F = 23.796, p < .001, partial η² = .488). 

**Table 2:** Tests of within-subjects’ contrasts after six months follow up

**Quality of life domain**	**f-value**	**P value**	**partial** η²
Physical health	72.381	0.001	0.743
Psychological health	39.812	0.001	0.614
Social relationships	23.827	0.001	0.488
Perception of life environment	23.796	0.001	0.488

## Discussion

Cocalear implants (CIs) have been shown to significantly improve the quality of life (QoL) in adults with severe to profound sensorineural hearing loss. According to the present study in which the WHOQOL questionnaires was conducted to assess the life quality in adult’s cochlear implant users, the participants experienced significant satisfaction in various aspects of physical health, psychological health, social relationships and perception of life environment. Examples of these situations are social communication, daily living activities, sleep and rest, mobility, work capacity or opportunities for acquiring new information and skills. The major strength of this study was the selection of adult participants with a uniform socioeconomic status and without any underlying medical conditions, which minimized the influence of potential confounding variables. In addition, a standardized Persian version of the Quality of Life questionnaire with Cronbach's alpha coefficients threshold of 0.70, indicating satisfactory internal consistency was used to assess the participants. Various studies have documented significant quality-of-life improvements in adults with post-lingual hearing loss following cochlear implantation. Notable improvements have been observed in communication abilities, psychological well-being, and social interactions. Additionally, factors such as perceived social support and the ability to understand speech over the telephone play crucial roles in determining post-implantation quality of life(6,13-15). 

A 2021 study involving 104 adults with cochlear implants reported the following findings: Before implantation, the overall quality of life averaged 0.51, with a standard deviation of approximately 45% of the mean. Participants demonstrated better functioning in the physical domain (mean: 0.50) compared to the psycho-social domain (mean: 0.27). The highest scores were observed in the dimensions of independent living, pain, and coping, while the lowest were in senses and mental health. The psycho-social super dimension showed the greatest variation, as indicated by the high ratio of standard deviation to the mean(14).

Following cochlear implantation, the overall quality of life improved to an average of 0.66, with reduced variability (standard deviation at 29% of the mean). Participants achieved the highest scores in the dimensions of independent living, self-worth, and coping, while mental health remained the lowest-scoring dimension. Statistically significant improvements were observed in nearly all dimensions of health-related quality of life, except for Pain. The dimensions of independent living, senses, mental health, happiness, coping, relationships, and self-worth, as well as both super dimensions, showed substantial improvement (p < 0.001). Overall, the study concluded that the quality of life significantly increased after cochlear implantation(14).

Significant improvements in speech perception and auditory comprehension in quiet and noisy environments after implantation are the most important results obtained from cochlear implantation(16).

Enhanced ability to engage in conversations reduces communication barriers, increasing independence and participation in daily activities. Some evidence suggests that cochlear implants may help slow the progression of cognitive decline in older adults by enhancing sensory input and encouraging social engagement (17-19).

Substantial gains in sentence recognition scores among adult CI users that lead to easier interpersonal interactions is another positive effect of cochlear implantation. Hearing loss often leads to withdrawal from social settings due to difficulty in following conversations. CIs help mitigate this by enabling individuals to feel more connected and engaged in group settings. Enhanced hearing fosters better communication with family and friends, positively influencing personal and professional relationships (20).

Several studies suggest that cochlear implants significantly decrease symptoms of depression and anxiety in adults with hearing loss, attributable to regained auditory function and improved social participation. Also, improved ability to participate in conversations and daily activities boosts confidence and overall self-perception (20-22). Cochlear implants may help slow the progression of cognitive decline in older adults by enhancing sensory input and encouraging social engagement. Many users report improved performance at work due to better communication, which can lead to career advancements. The ability to access auditory information more effectively often renews interest and capability in learning opportunities. The benefits of cochlear implants continue to grow with prolonged use as users adapt to the device and auditory environments.

Finally, it is necessary to indicate that while the majority of studies report positive outcomes of cochlear implantation, some factors can influence the degree of QoL improvement:

- Older adults and those with longer durations of hearing loss generally experience less benefits following implantation surgery.
- Active engagement in auditory and speech therapy is crucial for optimizing results.
- Also, some users experience technical challenges or discomfort, which may temporarily limit perceived benefits.

So, it is recommended that cochlear implantation in adults be performed shortly after the onset of deafness. Additionally, facilitating the patient's auditory comprehension should be pursued through adherence to rehabilitation programs. What is more, this study was conducted with a small sample size because according to the aforementioned criteria and after controlling for confounding variables, only this number of patients met the inclusion criteria for the study. In addition, only followed up with patients for a period of six months after surgery was carried out. Since it may not reflect long-term changes, future studies with extended follow-up are recommended. finally, it would be beneficial to pursue with a larger sample size and longer follow-up periods to optimize patient outcomes, and better understanding the factors influencing individual experiences. 

## Conclusion

Cochlear implants are transformative devices that can be effectively used not only in children but also in adults with severe to profound hearing loss. It has been well established that cochlear implantation, along with appropriate postoperative rehabilitation, significantly improves multiple domains of quality of life (QoL). The results of the present study demonstrated a marked sense of satisfaction among adult cochlear implant users across various aspects of physical health, psychological well-being, social relationships, and perception of their living environment. A major strength of this study was the inclusion of adults with uniform socioeconomic status and without underlying medical conditions, which helped minimize the influence of potential confounding variables. Moreover, the use of a standardized Persian version of the Quality-of-Life questionnaire with high internal validity ensured the reliability and cultural relevance of the findings. 
